# Supplementary material for: Structural transformation and political economy: A new approach to inclusive growth
Source: PLoS One. 2023 Aug 2;18(8):e0283731. doi: 10.1371/journal.pone.0283731 (PMC10395814; doi:10.1371/journal.pone.0283731)
Supplement: S1 Appendix — (DOC) [file pone.0283731.s001.doc]

**Appendix A:**

**Figure 1: Sectoral Employment and GDP Growth**


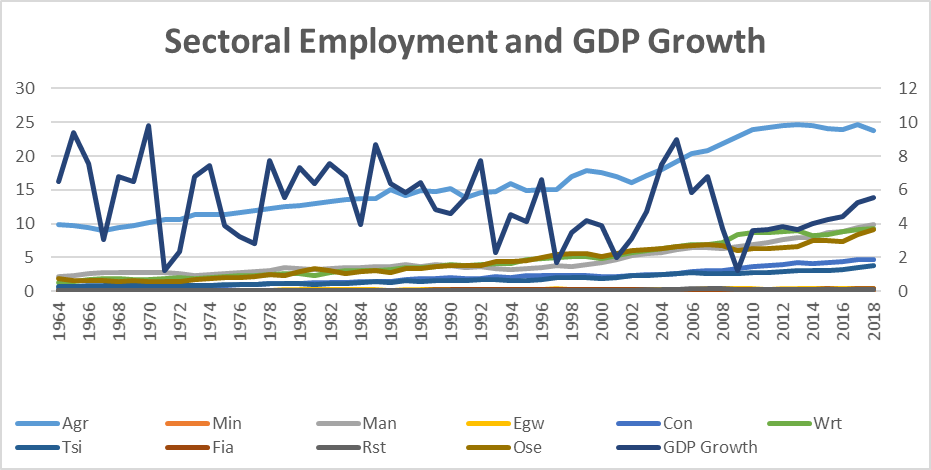
 Source: Authors' Estimation

**Figure 2: Employment Output Ratio And GDP Growth**


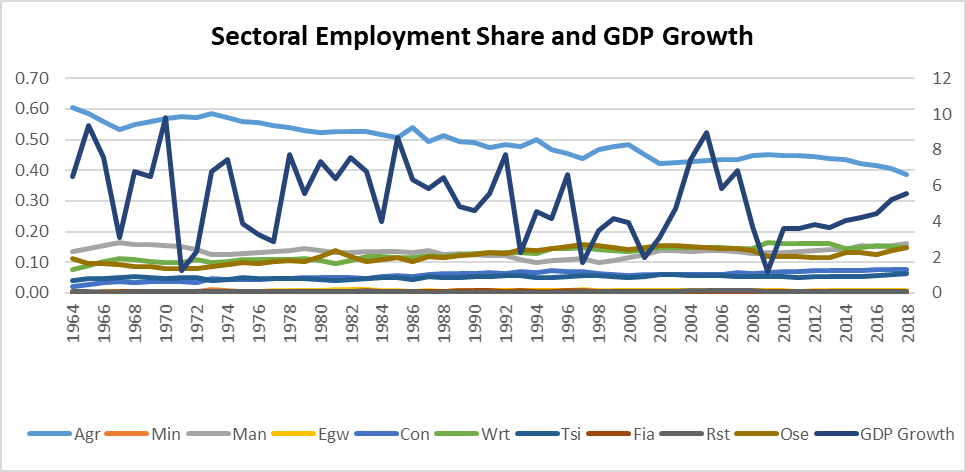
Source: Authors' Estimation

**Figure 3: Sectoral Employment and GDP Growth**


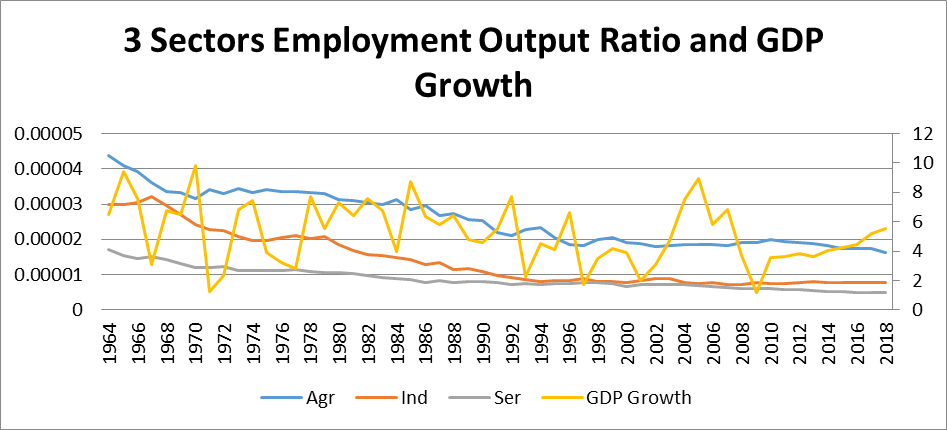
 Source: Authors' Estimation

**Figure 4: Agriculture Sector Employment and GDP Growth**


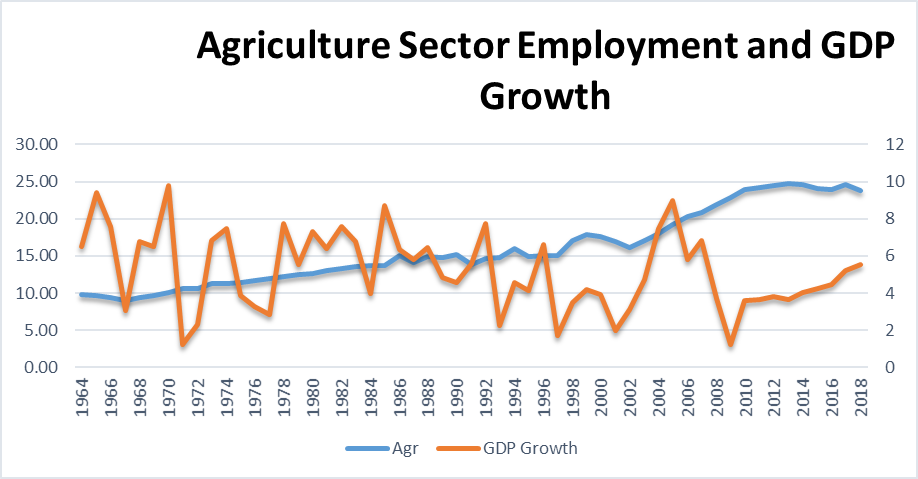
 Source: Authors' Estimation

**Figure 5: Agriculture Employment Output Ratio and GDP Growth**


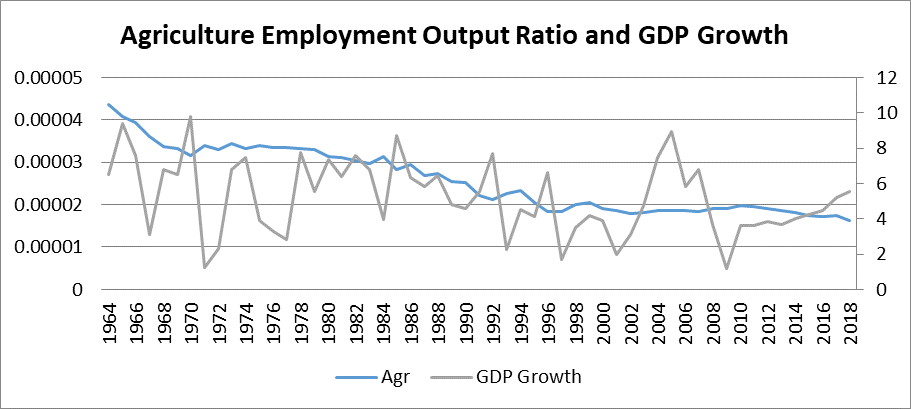
 Source: Authors' Estimation

**Figure 6: Industry Employment Output Ratio and GDP Growth**


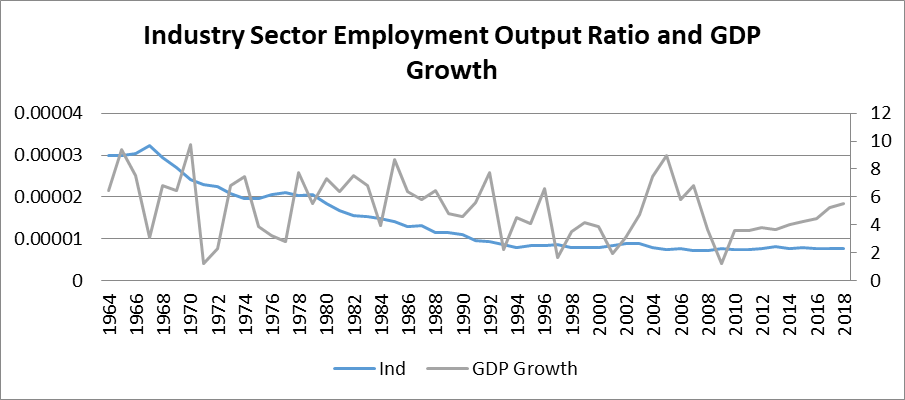
 Source: Authors' Estimation


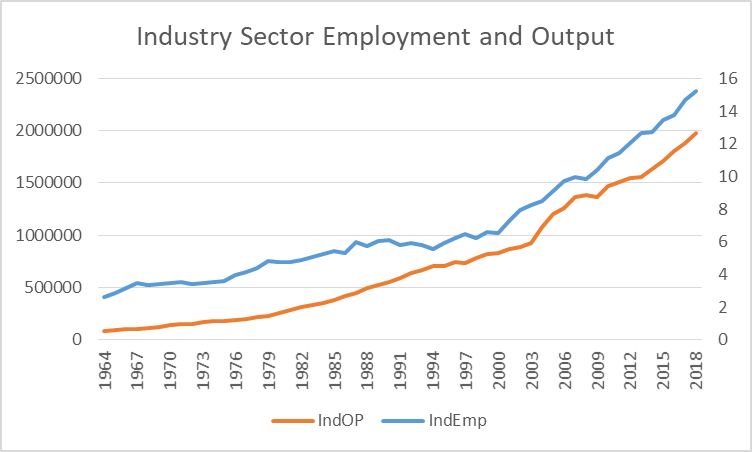


**Figure 7: Service Employment Output Ratio and GDP Growth**


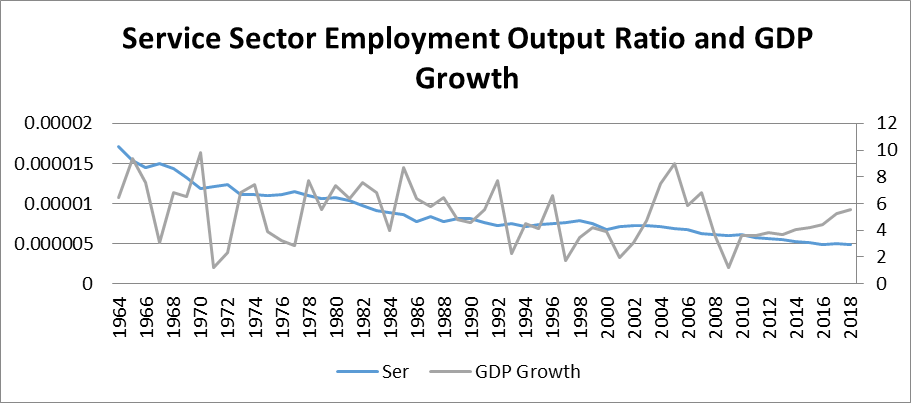


Source: Authors' Estimation
