# Supplementary material for: Structural transformation and political economy: A new approach to inclusive growth
Source: PLoS One. 2023 Aug 2;18(8):e0283731. doi: 10.1371/journal.pone.0283731 (PMC10395814; doi:10.1371/journal.pone.0283731)
Supplement: Attachment — Submitted filename: Response to Reviewers letter.doc [file pone.0283731.s002.doc]

**Response to Reviewers letter**

Reviewer #1: 1. Please rewrite abstract and elaborate more on the main findings of your analyses.
**Answer#1:** Accounting for the reviewer's comment, I am rewriting the abstract and also adding the main finding of the analysis

2. Describe your data and justify the time line of data selection in your write up.
**Answer#2:** The study took the data starting from 1964-67 till 2015-18, combine this complete time is spread over the 54 years of Pakistan's employment history. This study has collected the data of employed labour force through Labour Force Survey‘s (LFS) various issues and Pakistan Economic Survey which is published on the annual basis by Federal Bureau of Statistics, government of Pakistan.

3. Policy and recommendation section must include the main findings and their implication.

**Answer#3:** On the bases of the analysis and conclusions, some recommendations are added to the article.

Reviewer #2: I read the paper very carefully and topic is very interesting. The paper have many issues for example methodology is very poor and not written properly. It is very hard to extract information from the graphs given in the text. The paper is very much lengthy and its length can be reduced by merging the graph. I think authors need to employ latest econometric techniques.

**Answer#:** I have rewritten almost the whole paper and tried to reduce the all issue mentioned above.

Reviewer #3: This manuscript reports on “Structural Transformation and Political Economy: A New Approach to Inclusive Growth”. I want to suggest a few suggestions to improve the manuscript's quality and better readability.
1. The English language needs more work. There are many grammatical and typo mistakes in this manuscript. The paper needs to be edited by a native English speaker.

**Answer#:** removed the English language issue and delete typo error mistakes.

2. The abstract should state briefly the purpose of the research, the principal results, and major conclusions. An abstract is often presented separately from the article, so it must be able to stand alone.

**Answer#:** Accounting for the reviewer's comment, I am rewriting the abstract and also adding the main finding of the analysis

3. We prefer if you use the third person singular, instead of the first person singular or plural (e.g. "you, we, our").

**Answer#:** done

4. The major defect of this study is the debate or argument is not clearly stated in the introduction session. Hence, the contribution is weak in this manuscript. I would suggest the author enhance your theoretical discussion and arrives at your theoretical argument.

**Answer#:**

5. The authors made this study unnecessary long and vague. Just presenting some facts and figures are not enough.

**Answer#:** reduced the length of the article

6. The copy paste diagrams are not allowed and suitable references should be provided.

**Answer#:** All tables and figures are authors' estimated except the “Studies on the employment intensity of growth at sectoral level” table which the source is giving with the table.

7. Theoretical framework section is missing.

**Answer#:** In the methods section tried to explain the theoretical framework of the study

8. Elasticity method should be referenced.

**Answer#:** done
9. See lines 687-688: the authors mentioned “(the citation must be included here)”. Please be consistent.

**Answer#:** Dr. Faheem Jehanger Khan, https://pide.org.pk/blog/economic-growth/

10. Please make sure your conclusions' section underscore the scientific value added of your paper, and/or the applicability of your findings/results, as indicated previously. Please revise your conclusion part into more details. Basically, you should enhance your contributions, limitations, underscore the scientific value added of your paper, and/or the applicability of your findings/results and future study in this session.

**Answer#:** add all suggestions in the conclusion section

11. Notation" should be added to the article.

**Answer#:** done
12. What are the next policy implications?

**Answer#:** On the bases of the analysis and conclusions, some recommendations are added to the article.

13. What are the limitations and future research agenda?

**Answer#:** Due to a lack of information, this study employed the simple Elasticity technique, which involves first building an economic framework using all social, economic, and competitiveness factors, and then using that framework to examine the estimated elasticities.
